# The impact of educational attainment on the occurrence of gestational diabetes mellitus in two successive pregnancies of Finnish primiparous women: a population-based cohort study

**DOI:** 10.1007/s00592-020-01517-5

**Published:** 2020-04-02

**Authors:** Kristiina Rönö, Senja Masalin, Hannu Kautiainen, Mika Gissler, Johan Gunnar Eriksson, Merja Kristiina Laine

**Affiliations:** 1 Obstetrics and Gynecology, University of Helsinki and Helsinki University Hospital, Helsinki, Finland; 2 Folkhälsan Research Center, Helsinki, Finland; 3 Department of General Practice and Primary Health Care, University of Helsinki and Helsinki University Hospital, Helsinki, Finland; 4 Primary Health Care Unit, Kuopio University Hospital, Kuopio, Finland; 5 Information Services Department, Finnish Institute for Health and Welfare, Helsinki, Finland; 6 Department of Neurobiology, Care Sciences and Society, Karolinska Institute, Huddinge, Sweden; 7 Department of Obstetrics and Gynecology, Yong Loo Lin School of Medicine, National University of Singapore, Singapore, Singapore; 8 Agency for Science, Technology and Research (A*STAR), Singapore Institute for Clinical Sciences (SICS), Singapore, Singapore; 9 Vantaa Health Centre, Vantaa, Finland

**Keywords:** Educational status, Maternal, Diabetes, Gestational, Pregnancy, Prevalence, Recurrence

## Abstract

**Aims:**

To assess the impact of educational attainment on the occurrence and recurrence of gestational diabetes mellitus (GDM) in two successive pregnancies in primiparous women.

**Methods:**

This is a population-based observational cohort study including all 2347 Finnish women without previously diagnosed diabetes, aged ≥ 20 years from the city of Vantaa, Finland, who gave birth to their first and second child between 2009 and 2015. National registries provided data on study participants. We divided the population into four groups according to the presence of GDM in the two pregnancies (GDM−/−, *n* = 1820; GDM−/+, *n* = 223; GDM+/−, *n* = 113; GDM+/+, *n* = 191).

**Results:**

The occurrence of GDM in the first pregnancy was 13.0% (*n* = 304) and 17.6% (*n* = 414) in the second. The recurrence rate of GDM was 62.8%. The four groups did not differ in relation to educational attainment (*p* = 0.11). In multinomial regression analysis, educational attainment protected from GDM in the second pregnancy [relative risk ratio 0.93 (95% confidence interval (CI) 0.86–0.99) per year of schooling for being GDM−/+ compared with GDM−/−]. In multivariate logistics models, prepregnancy body mass index at the first pregnancy [odds ratio (OR) 1.53 per 1-standard deviation (SD) (95% CI 1.22–1.91)], first-born birth weight *z*-score [OR 1.30 per 1-SD (95% CI 1.00–1.67)], and inter-pregnancy weight change [OR 1.66 per 1-SD (95% CI 1.27–2.16)], but not educational attainment, predicted recurrence of GDM.

**Conclusions:**

The recurrence rate of GDM was high. Education protected from novel GDM in the second pregnancy, but was not associated with GDM recurrence.

## Introduction

Gestational diabetes mellitus (GDM) is a serious and increasing public health issue and a common pregnancy complication associated with adverse short- and long-term health outcomes for the woman and her offspring [1–3]. The incidence of GDM worldwide varies from 2 to 25% [4]. Noteworthy is that the recurrence of GDM is high and likewise varies widely from 30 to 84% [5].

Advancing age [6, 7], a family history of diabetes [7], and ethnicity of a non-Anglo-European decent [8] are well-established risk factors for GDM. Multiparity is also associated with an increased risk of GDM [6], although advancing maternal age and weight gain both during and between pregnancies seem to mediate the effect [9]. Additionally, both ethnicity and parity have a significant effect on the recurrence rate of GDM. The recurrence rates are lowest in primiparous women and in non-Hispanic whites, whereas the rates are highest in multiparous women and in women of other ethnicities [10]. Modifiable risk factors for the occurrence and recurrence of GDM are mostly lifestyle related, such as degree of adiposity [11, 12], excessive gestational weight gain [13], and inter-pregnancy weight gain [14, 15].

Higher educational attainment, reflecting the knowledge-related assets of an individual [16], is usually associated with better health outcomes in the general population [17]. Findings concerning the impact of education on GDM risk are, however, inconclusive. Some studies have reported an inverse relationship between these two factors [18, 19], while the same relationship has not been observed in other studies [20, 21]. To the best of our knowledge, no previous studies have focused on educational attainment and the risk of occurrence and recurrence of GDM in European primiparous women.

In 2016, we initiated a follow-up cohort study to assess the long-term consequences of gestational glucose intolerance on the health of women and their offspring in the city of Vantaa, Finland. We have previously shown that within this cohort, income and education are inversely associated with the occurrence of GDM in primiparous women [22]. This paper, assessing a subgroup from the prior study, aims to evaluate further the influence of maternal educational attainment on the presence of GDM in the first two pregnancies leading to delivery, also taking into account traditional risk factors like maternal age and adiposity for GDM.

## Participants and methods

This study is an observational cohort study in the city of Vantaa, Finland. The city of Vantaa with 220 000 inhabitants is the fourth largest city in Finland. The study participants (*N* = 2347) consist of all those Finnish women (i.e., women born in Finland with Finnish or Swedish as native language), aged 20 years or older, from the city of Vantaa, who gave birth to their first and second live singleton child between the 1st of January 2009 and the 31st of December 2015. We excluded women with preexisting diabetes mellitus based on data obtained from the Finnish Social Insurance Institution.

The Finnish Medical Birth Register, maintained by the Finnish Institute for Health and Welfare, receives the information on all live births and stillbirths from 22 weeks of gestation or a birth weight of 500 g onward. From this source, we obtained data on deliveries, maternal age, GDM diagnoses, antenatal hospitalization due to hypertensive disorders during pregnancy (including ICD-10 codes O10, O13, and O14), prepregnancy height and weight, number of previous pregnancies (including miscarriages, induced abortions, and ectopic pregnancies), smoking during pregnancy, cohabitation status, and use of any infertility treatment [23]. We additionally collected supplemental data on missing information on GDM, height, and weight from individual patient healthcare records. We obtained data on educational attainment as years of schooling from Statistics Finland. Nine to ten years of school corresponds to basic (compulsory) education; 11–14 years of school corresponds to upper secondary education or post-secondary non-tertiary education; 15–16 years of school corresponds to bachelor’s or equivalent education, and 17 years or more of school corresponds to master’s, doctoral, or equivalent education. The Finnish Tax Administration provided data on maternal annual taxable income before the first pregnancy.

In Finland, GDM is screened in public antenatal clinics in primary healthcare centers according to the Finnish Current Care Guidelines for GDM [24]. Since 2008, GDM has been screened using a 75-g 2-h oral glucose tolerance test between 24 and 28 weeks of gestation in all pregnant women, with the exception of those who are at low risk, i.e., nulliparous women aged < 25 years, with a body mass index (BMI) 18.5–24.9 kg/m^2^, and without a first-degree family history of diabetes; or multiparous women aged < 40 years, with a BMI < 25 kg/m^2^, and without prior GDM or previous offspring macrosomia. Women at the highest risk (e.g., with a BMI ≥ 35 kg/m^2^, prior GDM, glycosuria, first- or second-degree family member with type 2 diabetes, continuous use of oral corticosteroids, or polycystic ovary syndrome) are tested for the first time already at 12–16 weeks of gestation, and if the results are negative, the test is repeated between 24 and 28 weeks of gestation. One or more pathological glucose value in the oral glucose tolerance test with the following diagnostic thresholds: fasting plasma glucose ≥ 5.3 mmol/L, 1-h glucose ≥ 10.0 mmol/L, and 2-h glucose ≥ 8.6 mmol/L lead to a diagnosis of GDM.

**Fig. 1:**
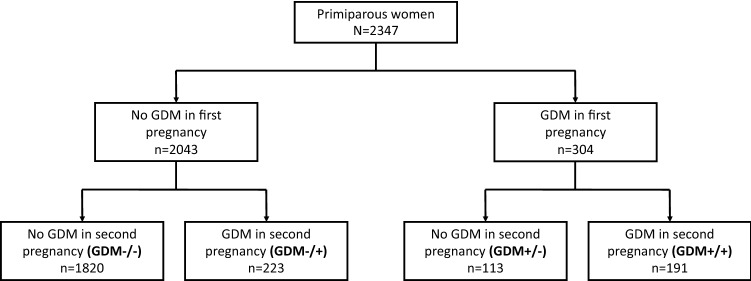
Flowchart of the first and the second pregnancy of 2347 Finnish primiparous women, aged 20 years or older, from the city of Vantaa, Finland, giving birth between 2009 and 2015, according to the occurrence of gestational diabetes (GDM)

We divided the study population into four groups according to the occurrence of GDM in the first two pregnancies leading to delivery: no GDM (GDM−/−), GDM only in the second pregnancy (GDM−/+), GDM only in the first pregnancy (GDM+/−), or GDM in both pregnancies (GDM+/+) (Fig. 1).

### Statistical analysis

Data are presented as means with standard deviations (SD) for continuous variables, or as counts with percentages for categorical variables. We compared differences of participant characteristics between subgroups of participants (according to the occurrence of GDM in the two successive pregnancies) using analysis of variance (ANOVA), with Hommel’s multiple comparison procedure to correct significance levels for post hoc testing. A multinomial logistic regression analysis was applied to assess the relative risk ratios (RRRs) of characteristics at the first pregnancy for the presence of GDM in the two successive pregnancies, with the women with no GDM (GDM−/−), as a reference group. A multivariate logistic regression model was used to examine the predictive odds ratios (OR) of participant characteristics on the risk of recurrence of GDM. Stata 15.1 (StataCorp LP; College Station, Texas, USA) statistical package was used for the analyses.

## Results

The mean age of the women in the study population at the time of the first delivery was 28.3 (SD 4.3) years. The mean length of education in the cohort was 13.8 (SD 2.5, range 9–22) years, and mean BMI before the first pregnancy 23.9 (SD 4.4) kg/m^2^. The correlation between length of education and BMI was − 0.04 (− 0.08 to − 0.01). The occurrence of GDM was 13.0% (*n* = 304) in the first pregnancy and 17.6% (*n* = 404) in the second pregnancy. The recurrence rate of GDM was 62.8% (*n* = 191/304). The mean interval between the two successive deliveries was 2.4 (SD 0.9) years.

Table 1 presents the characteristics of the study participants before their first pregnancy and between the two successive pregnancies according to the presence of GDM. Women in the GDM−/+ group gained 5.0 (SD 6.4) kg of weight between the two pregnancies, while the ones in the GDM+/− group lost 0.5 (SD 5.6) kg of weight between the two pregnancies (*p* < 0.001 between groups) (Table 1). The results concerning inter-pregnancy weight change remained the same after adjustments for the following characteristics at the first pregnancy: age, smoking, educational attainment, prepregnancy BMI, use of any fertility treatment, and hypertensive disorder during pregnancy.

**Table 1:** Participant characteristics of 2347 Finnish primiparous women, aged 20 years or more, and without previously diagnosed diabetes mellitus, according to the occurrence of gestational diabetes (GDM) in two successive pregnancies

	No GDM in first pregnancy		GDM in first pregnancy		
	No GDM in second pregnancy (GDM−/−) *N* = 1820	GDM in second pregnancy (GDM−/+) *N* = 223	No GDM in second pregnancy (GDM+/−) *N* = 113	GDM in second pregnancy (GDM+/+) *N* = 191	*p* value between groups (multiple comparison)^a^
*Characteristics at first pregnancy*					
Age (years), mean (SD)	28.1 (4.3)	28.7 (4.1)	28.5 (4.3)	29.3 (4.2)	0.003 [1/4]
Cohabiting, *n* (%)	1510 (83)	197 (88)	96 (85)	159 (83)	0.23
Smokers, *n* (%)	255 (14)	41 (18)	22 (19)	32 (17)	0.13
Years of schooling, mean (SD)	13.9 (2.5)	13.5 (2.5)	13.5 (2.4)	13.7 (2.4)	0.11
Annual income (1000 EUR), mean (SD)	26.9 (12.9)	26.6 (11.7)	26.8 (12.7)	27.1 (13.1)	0.96
Height (cm), mean (SD)	166 (6)	165 (6)	165 (6)	165 (6)	0.22
Weight (kg), mean (SD)	64 (12)	70 (14)	69 (14)	76 (17)	< 0.001 [1/2, 1/3, 1/4, 2/4, 3/4]
Prepregnancy BMI (kg/m^2^), mean (SD)	23.2 (3.8)	25.3 (4.6)	25.3 (5.1)	27.7 (5.6)	< 0.001 [1/2, 1/3, 1/4, 2/4, 3/4]
Prepregnancy obesity (BMI ≥30 kg/m^2^), *n* (%)	114 (6)	35 (16)	20 (18)	62 (32)	< 0.001 [1/2, 1/3, 1/4, 2/4, 3/4]
Previous pregnancies^b^, *n* (%)					0.86
None	1494 (82)	180 (81)	87 (77)	155 (81)	
1	237 (13)	29 (13)	19 (17)	26 (14)	
≥2	89 (5)	14 (6)	7 (6)	10 (5)	
Any fertility treatment, *n* (%)	127 (7)	19 (9)	16 (14)	25 (13)	0.002 [1/3, 1/4]
Hypertensive disorder of pregnancy^c^, *n* (%)	88 (5)	11 (5)	9 (8)	21 (11)	0.003 [1/4]
Birth weight (*z*-score), mean (SD)	− 0,06 (0,97)	0,23 (1,03)	− 0,06 (1,11)	0,31 (1,07)	0.001 [1/2 1/4, 2/3,4/3]
*Characteristics between pregnancies*					
Weight change between pregnancies (kg), mean (SD)	1.4 (5.0)	5.0 (6.4)	− 0.5 (5.6)	2.2 (6.3)	< 0.001 [1/2, 1/3, 2/3, 2/4, 3/4]
Time between deliveries (years), mean (SD)	2.4 (0.9)	2.5 (1.0)	2.3 (0.9)	2.4 (1.1)	0.53

^a^Hommel’s multiple comparison procedure was used to correct significance levels for post hoc testing (*p* < 0.05)

^b^Miscarriages, induced abortions, and ectopic pregnancies

^c^Antenatal hospitalization with ICD-10 codes O10, O13, or O14

In a multinomial logistic regression model, higher educational attainment decreased the relative risk ratio (RRR) for the presence/occurrence of GDM in the second pregnancy only (GDM−/+), when compared to women with no GDM (GDM−/−) (*p* = 0.016) (Table 2). A similar, but nonsignificant trend was seen in GDM+/− and GDM +/+ groups, with the GDM−/− group as reference.

**Table 2:** Relative risk ratio (RRR)^a^ of patient characteristics at the first pregnancy to predict risk of gestational diabetes (GDM) only in the first pregnancy, only in the second pregnancy, and in both pregnancies

	Occurrence of gestational diabetes					
	Only 1st pregnancy^b^ (GDM+/−) *N* = 113		Only 2nd pregnancy^b^ (GDM−/+) *N* = 223		Both pregnancies^b^ (GDM+/+) *N* = 191	
	RRR (95% CI)	*p* value	RRR (95% CI)	*p* value	RRR (95% CI)	*p* value
Age	1.03 (0.98–1.09)	0.21	1.06 (1.02–1.10)	0.004	1.07 (1.03–1.12)	< 0.001
Cohabiting	1.18 (0.69–2.03)	0.54	1.57 (1.02–2.44)	0.041	0.90 (0.59–1.38)	0.64
Smoking	1.40 (0.86–2.46)	0.17	1.40 (0.94–2.08)	0.98	1.24 (0.79–1.96)	0.35
Years of schooling	0.93 (0.85–1.03)	0.12	0.92 (0.86–0.99)	0.016	0.95 (0.89–1.03)	0.22
BMI (kg/m^2^)	1.12 (1.07–1.16)	< 0.001	1.12 (1.08–1.15)	< 0.001	1.21 (1.17–1.24)	< 0.001
Any fertility treatment	2.34 (1.31–4.18)	0.004	1.25 (0.74–2.10)	0.40	2.04 (1.24–3.34)	0.005
Hypertensive disorder^c^	–	–	0.83 (0.43–1.59)	0.57	1.75 (1.02–3.02)	0.043

^a^Multinominal logistic regression model was used to assess the RRR

^b^Women having GDM in neither pregnancy (GDM−/−; *n* = 1820) are the reference group for the model

^c^Hypertensive disorder in the first pregnancy (antenatal hospitalization with ICD-10 codes O10, O13, and O14)

**Fig. 2:**
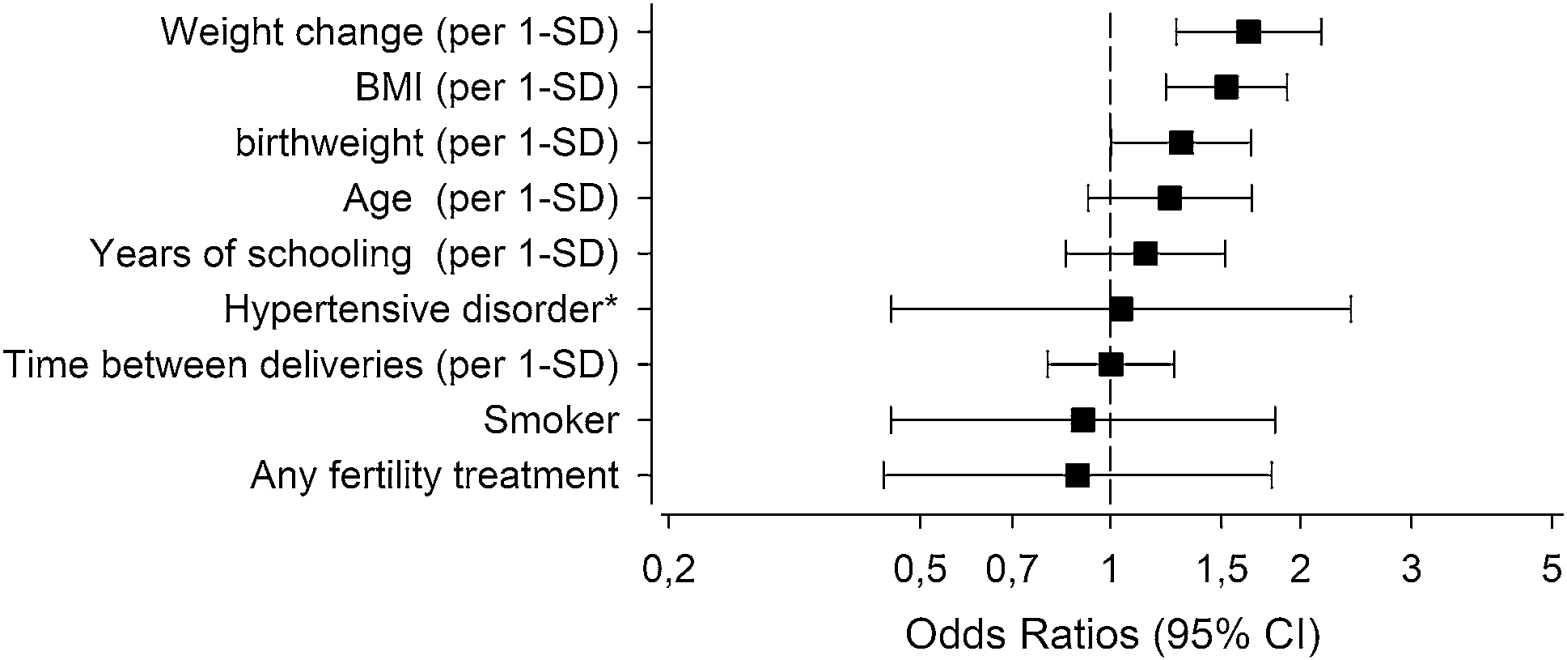
Risk of recurrence of gestational diabetes according to participant characteristics at the first pregnancy (prepregnancy body mass index [BMI], birth weight of the first child, maternal age at delivery, years of schooling, and *antenatal hospitalization in the first pregnancy due to hypertension [ICD-10 codes O10, O13, and O14], and smoking) and between pregnancies (weight change, and time as years). Multivariate logistic regression model was used to assess the risk

In a multivariate logistic regression model, the independent predictive factors for recurrence of GDM were prepregnancy BMI at the first pregnancy, birth weight *z*-score of the first child, and inter-pregnancy weight change (Fig. 2). The OR for recurrence of GDM was 1.53 (95% CI 1.22–1.91]) for each 1-SD increase in prepregnancy BMI at the first pregnancy, 1.30 (95% CI 1.00–1.67) for each 1-SD increase in birth weight *z*-score of the first child, and 1.66 (95% CI 1.27–2.16) for each 1-SD increase in weight change between pregnancies.

## Discussion

The occurrence of GDM in the first pregnancy was 13.0%, and 17.6% in the second pregnancy. The recurrence of GDM was high, 63%. We detected a trend of higher education protecting against the presence of GDM in either or both of the two successive pregnancies in our cohort of Finnish primiparous women. The protective effect, however, was significant only when comparing women with GDM present only in the second pregnancy (GDM−/+) with women without GDM in neither of the pregnancies (GDM −/−). Maternal educational attainment did not, interestingly enough, influence the recurrence of GDM. Independent predictors of recurrence of GDM were BMI before the first pregnancy, birth weight of the first child, and inter-pregnancy weight change.

Our study has several strengths: The study cohort is comprehensive; all Finnish women from the city of Vantaa, aged 20 years or older, without preexisting diabetes, and who delivered for the first and second time during a 7-year study period, were included in the study. We included only primiparous women to exclude the confounding effects of previous GDM or parity on the risk of GDM. The quality of the Finnish Medical Birth Register is considered good [25], and individual patient healthcare records provided an additional supplement for missing data. The national diagnostic criteria for GDM remained the same during the whole study period and are based on a 75-g standard 2-h oral glucose tolerance test. Further, educational attainment and annual maternal taxable income were not self-reported, rather based on data obtained from Statistics Finland and The Finnish Tax Administration, respectively.

Our study also has some limitations, including the lack of some well-known risk factors for GDM such as family history of diabetes and gestational weight gain. Furthermore, some of the women may have had an unrecognized diabetes before or between the pregnancies and might have been misclassified as having GDM. As this is an observational register-based cohort study, we also lack data on lifestyle-related factors including diet, physical activity, and sleeping patterns. Lastly, the study participants were all Finnish women, mainly of European ancestry, limiting the generalization of the results globally.

The occurrence of GDM in our cohort is in line with the nationwide Finnish occurrence, including all women regardless of parity, that increased from 9 to 16% during the study period between 2009 and 2016 [23]. The recurrence rate of over 60% of GDM can be considered high—especially as non-Hispanic whites (recurrence rate of 35% compared with 56% in other ethnicities), and primiparous women (recurrence rate of 40% compared with 73% in multiparous women) are regarded as having the lowest risk of recurrence [10]. Most studies have reported lower recurrence rates (38–47%) of GDM in general [14, 26–29]. Disparities between study populations including ethnicity, genetic predisposition to both T2D and GDM, and the screening methods and diagnostic criteria for GDM can probably explain the varying observations regarding recurrence rates of GDM. Further, the number of prior pregnancies complicated by GDM has an impact on the GDM recurrence. According to a Canadian study, women who had GDM in an index pregnancy had a recurrence rate of GDM of 72% in the second subsequent pregnancy, if the first subsequent pregnancy was also affected [30]. If the first subsequent pregnancy was not affected by GDM, the recurrence rate in the second subsequent pregnancy was only 22% [30].

In our study cohort, there was a trend of education having a protective effect on GDM. The finding was, however, significant only when comparing women with GDM only in the second pregnancy (GDM−/+) with women without GDM in neither pregnancy (GDM−/−). In our prior study, including a larger cohort of primiparous women, incidence of GDM showed an inverse association with educational attainment [22]. Further, there was no association between educational attainment and recurrence of GDM in the current study. Although some prior studies, including both primiparous and multiparous women, have found low-educated women having a higher risk of GDM than high-educated women [18, 19, 22]. However, in some other studies, no such association between education and risk of GDM has been detected [20, 21]. The use of various criteria for educational attainment, as well as sample size and other factors related to study design, might at least partly explain these conflicting findings. Even less is known of the impact of education on the risk of recurrence of GDM [29, 31].

GDM is a heterogeneous disease with several underlying causes such as inadequate insulin secretion from pancreatic β-cells, insulin resistance, and genetic susceptibility [32, 33]. The dysfunction of pancreatic β-cells is probably not limited only to pregnancy, as it is also evident before pregnancy and during the postpartum period. Women who develop GDM seem additionally to be more insulin resistant already before pregnancy [34, 35]. These factors may at least partly explain the high recurrence of GDM [32, 36, 37]. Factors increasing insulin resistance include high childbearing age, chronic low-grade inflammation due to obesity-induced lipid accumulation, and insulin-desensitizing effects of the hormonal products of the placenta [37, 38].

Both a higher BMI and an advancing maternal age are traditional recognized risk factors for occurrence of GDM [6, 39]. In our cohort, a higher prepregnancy BMI predicted GDM in either pregnancy, or in both of them, compared with women who did not develop GDM at all. Our finding of an increasing BMI having an independent predictive value for GDM recurrence is also supported by findings in the literature [40]. Both a higher maternal BMI at the index pregnancy [11, 12, 41] and an increase in weight before the subsequent pregnancy [30] are previously recognized risk factors for GDM recurrence.

Our observation that inter-pregnancy weight gain independently increases the risk of GDM recurrence is also in line with several other studies [11, 12, 15, 28, 42]. In a meta-analysis by Schwartz and colleagues, weight gain between pregnancies was reported to have the largest effect size of studied risk factors for GDM recurrence [40]. Weight gain between pregnancies increases the level of insulin resistance, which, in turn, may contribute to β-cell exhaustion and thus, elevate the risk of GDM in the subsequent pregnancy [5, 14]. Weight loss between pregnancies may improve insulin sensitivity as well as β-cell function and thus, decrease the risk of GDM in the subsequent pregnancy [14]. According to a large cohort study in the USA, a decrease in BMI between pregnancies may act as a protective factor for GDM especially in overweight and obese women [14].

Prior infant birth weight [30], macrosomia [29], and LGA offspring [27, 41] have been shown to be predictive for recurrence of GDM in some studies. Our observation concerning the association of birth weight of the first child and risk of GDM in the subsequent pregnancy is in line with these findings.

Prior findings in the literature additionally suggest advancing age being a risk factor for GDM recurrence [11, 27]. In our cohort, the age of the mother had, however, no independent predictive role for the recurrence. Compared with women who did not develop GDM in either pregnancy, advancing age did only predict the occurrence of GDM in the second pregnancy, independent of having GDM in the first pregnancy. According to some former findings, both shorter [12, 29] and longer [26, 27] inter-pregnancy intervals have shown an association with an increased risk of GDM recurrence. We did, nonetheless, observe no such association in our study population, which is in line with a recent meta-analysis [40].

Although hypertensive disorders in the index pregnancy have also been associated with an increased risk of GDM recurrence [27], we did not detect such an association in our study. The sample size in our study population was, however, rather small to assess the impact of hypertensive disorders, as displayed by large confidence intervals in the model.

According to the findings in our cohort, use of infertility treatment was more common in the first pregnancy for women who also developed GDM in the same pregnancy (GDM+/− and GDM+/+), compared with women who did not develop GDM in either pregnancy (GDM−/−). The finding is not surprising, as the risk of GDM has been shown to be increased in pregnancies with assisted reproduction technology treatments [43].

GDM and its recurrence are serious public health concerns that have implications for the health of women and their offspring over generations. Recurrence of GDM seems to increase the risk of developing diabetes later in life [44], but otherwise little is known about the impact of recurrence of GDM on maternal long-term health [45]. The healthcare system should ideally identify women with elevated risk of GDM already before their first pregnancy, as many of the factors such as an underlying insulin resistance and a dysfunction of pancreatic β-cells that might lead to development of GDM, exist already before conception. Further studies are required in order to evaluate how to reduce the risk of GDM recurrence.

## Conclusion

According to our study findings, education protects from a novel occurrence of GDM in the second pregnancy, when the first pregnancy has not been complicated by GDM. Maternal educational attainment seems to have no association, however, with the risk of GDM recurrence. Overall, the recurrence of GDM was high in our cohort. Our results suggest that maternal inter-pregnancy weight gain and a higher BMI before the first pregnancy, both factors being lifestyle related and modifiable, are the most important independent predictors for GDM recurrence in primiparous women.

## Data Availability

Data cannot be shared for both legal and ethical reasons. Data from the Finnish Institute for Health and Welfare, Statistics Finland, and the Finnish Social Insurance Institution can only be used for the purpose stated in the license granted, scientific research on society by the license applicant, and can therefore not be shared with third parties.
